# Feasibility, acceptability and effectiveness of a culturally informed intervention to decrease stress and promote well-being in reservation-based Native American Head Start teachers

**DOI:** 10.1186/s12889-023-16913-z

**Published:** 2023-10-25

**Authors:** Deborah H. Wilson, Danielle German, Adrian Ricker, Hilary Gourneau, Ginger C. Hanson, Justin Mayhew, Teresa N. Brockie, Michelle Sarche

**Affiliations:** 1 School of Nursing, Johns Hopkins University, 525 N Wolfe St., Baltimore, MD 21205 USA; 2 Auckland University of Technology School of Clinical Sciences, 90 Akoranga Drive, Northcote, Auckland, 0627 New Zealand; 3 Department of Health Behavior and Society, Johns Hopkins University, 624 N Broadway, Baltimore, MD 21205 USA; 4 Fort Peck Tribes Head Start, 409 G St, W Poplar, MT 59255, USA; 5 Center for Indigenous Health, Johns Hopkins Bloomberg School of Public Health, 415 N. Washington Street, Baltimore, MD 21231 USA; 6 Colorado School of Public Health, Centers for American Indian and Alaska Native Health, University of Colorado Anschutz Medical Campus, Mail Stop, 13001 E 17th Pl B119, Aurora, CO 80045 USA

**Keywords:** Native American, Head Start, Intervention implementation, Mental health, CBPR, Mixed methods, Feasibility study, Stress, Well-being

## Abstract

**Background:**

While benefiting from strong cultural ties to family, land and culture Native Americans residing on reservations experience psychological distress at rates 2.5 times that of the general population. Treatment utilization for psychological health in reservation-based communities is low with access to culturally appropriate care lacking. Evidence suggests that for mental health treatment, Native Americans prefer culturally informed care that respects Native perspectives on health and well-being.

**Methods:**

To decrease stress and promote well-being in tribal Head Start teachers we adapted and implemented a culturally focused intervention within a community-based participatory research framework using mixed methods. Feasibility and acceptability of the adapted 5-session curriculum was tested in a single arm intervention study with a sample of 18 teachers on the Fort Peck Reservation. Participants completed surveys at baseline and upon completion of the intervention. Within session observations and two post-intervention focus groups (*n* = 8, *n* = 10) were conducted to elaborate and explain the quantitative results eliciting participant experience of intervention effectiveness and feasibility, acceptably and appropriateness. Implementation outcomes were assessed quantitatively using the Acceptability of Intervention, Intervention Appropriateness, and Feasibility of Intervention measures.

**Results:**

Quantitively, attendance rate overall was 93% with no dropouts. Pretest/posttest surveys were analyzed using t-tests and Hedges g to measure effect size. Contrary to our hypothesis, self-perceived stress showed a small positive effect size, indicating that participants were more stressed post intervention. However, depression decreased, with tribal identity and resilience showing positive effect sizes. Content analysis for the qualitative data collected within session observations and post intervention focus groups revealed how lifetime traumas were affecting participants, providing some explanation for the increase in stress. Teachers reported that the sessions helped their psychological health and well-being, supporting feasibility of future interventions. Acceptability scored highest with a mean (SD) of 4.25 (.84) out of 5, appropriateness 4.18 (.86) and feasibility 4.06 (.96) supporting intervention to be acceptable, appropriate, and feasible.

**Conclusion:**

Utilizing a culturally based intervention to buffer stress and support the well-being of reservation-based teachers showed promise in helping them recognize their cultural strengths, stress, and need for ongoing support. Implementation outcomes show that intervention scale-out is feasible.

## Background

Native Americans comprise 1.7% of the United States (U.S.) population with 22% residing on geographically isolated reservations [1]. The term American Indian/Alaska Native is used by the Federal Government and U.S. Census Bureau. For this publication, however, the term “Native American” will be used throughout. A federal Native American reservation is land reserved for tribes as specified in treaties with the U.S. Federal Government which holds title to the land in trust on behalf of the tribe. While some reservations are located near a tribe’s original land base, most reservations were created to forcibly relocate Native Americans from their original homelands to free that land for colonial settlement, resource extraction, or other use [2].

Today, reservation-based tribes are sovereign with the right to self-government; tribal governments having jurisdiction over reservation lands, not the state or Federal Government [3]. However, this self-governance is hampered by tribal communities still existing within a federal system that has historically undermined their self-determination [4]. Federally recognized tribes receive health and education services as a treaty right through the federally funded Indian Health Service (I.H.S). Funding for the I.H.S. and other treaty rights to which tribes are entitled (e.g., education), however, is at the discretion of Congress. Funding is thus subject to political influence leaving the I.H.S and other programs woefully underfunded, thus driving the severe health disparities that Native Americans face [5].

With rich cultural heritages, traditions, community and familial connections, and languages, Native Americans have demonstrated strength and perseverance despite the historical traumas of colonization, genocide, broken treaties, forced relocation to reservations as well as contemporary racism and discrimination [6]. However, the effects of these intergenerational traumas can be seen in the high rates of health disparities. On reservations, poverty, lack of access to healthy foods, and under-resourced health care and schools, place Native Americans at risk for high rates of morbidity and mortality. For example, Native Americans experience adverse childhood experiences at 2.5 times the rate of the general population and are more likely to experience psychological stress and mental health issues such as post-traumatic stress disorder, substance use and suicide [7, 8]. According to the National Center for Health Statistics (2019) Native Americans report experiencing serious psychological distress 2.5 times more than the general population [9].

Compounding these alarming rates of psychological distress, treatment utilization –both biomedical and traditional – for mental health in reservation based Native American communities is low [10, 11]. Reasons range from lack of providers due to geographic isolation, providers who are not Native, transportation, or insurance, as well as stigma and concerns about privacy [10–12]. There is growing evidence that for mental health treatment, Native Americans would prefer a Native provider or culturally informed care that respects Native perspectives on health, healing, and well-being [10, 11, 13, 14]. Further, with health disparities such as depression, anxiety and substance use affecting Native American communities being connected to the historical and contemporary traumas they have endured, there is growing interest in behavioral health interventions that emphasize “culture as treatment” [10, 15, 16]. Culture as treatment is defined as the inclusion of traditional healing practices, cultural values, and messaging to replace mainstream models of health and policy intervention. Culture as treatment emphasizes cultural buffers such as language revitalization, positive tribal identity, and communal mastery to promote well-being and bolster individual and collective strengths [10, 15–19]. Participating in cultural activities such as smudging has also been shown to enhance a sense of self-worth, dignity, and connection with one’s culture. Smudging is a traditional way to remove negative thoughts, feelings, and promote positive energy [20]. Furthermore, evidence suggests that attention to cultural norms, practices and knowledge when adapting an intervention for a Native American context can contribute to the sustainability and acceptance of the intervention—something that is often overlooked when implementing public health interventions [21, 22].

The Fort Peck Reservation in northwestern Montana struggles with many disparities. A 2016 community health assessment reported 36% of households with children under 18 living in poverty compared to 20% for the rest of the state, and median household incomes of $34,345 compared to $46,766 for the rest of Montana [23]. The average mental health days reported in Fort Peck was 4.4 compared to 3.4 among other Montanans, exacerbated by the ratio of mental health providers to patients of 1 to 1,030 versus 1 to 399 across the rest of the state. The primary health care ratio is 1 provider to 5,563 patients versus 1 to 1,312, respectively [23]. This lack of providers, a chronically underfunded I.H.S., and issues with insurance, poverty and stigma hindering attempts to access care typifies Fort Peck as a reservation that needs quality and culturally sensitive psychological and behavioral health care.

Within this broader context, the Fort Peck tribe operates a Head Start program which provides early education services to the reservation’s preschool aged children and families. Head Start is designed to help break the cycle of poverty and provide children ages three to five from low-income families with a program that meets their emotional, social, health, nutritional, and psychological needs [24]. It is the largest federally funded early childhood education program in the U.S. The program is administered by the Administration for Children and Families (ACF), Office of Head Start (OHS) in12 regions [24]. Regions 1-X are geographically defined, whereas Regions XI and XII are defined by the communities they serve. Region X1 programs are those run by federally recognized Native American/Alaska Native tribes. While there is no specific data on Fort Peck Head Start teachers their administration informed the researchers that teachers report many of the children exhibit signs of depression or behavior problems, and that they feel a responsibility to address these issues despite little support from specialist services so that the children may grow into healthy adults (personal communication C. Wetsit, October 2021). The reasons given for the lack of specialists to support the teachers and children include the remote location of the reservation, long waiting lists for services and children being bumped from services because of others in crisis such as suicides so the burden falls on the Head Start teachers to manage these children. Existing Head Start organizational support for teachers on the reservation is limited to trainings on working with autistic children and trauma-informed care.

### Preliminary work

Due to there being limited data on the Fort Peck Head Start teachers experience and needs we spent time conducting qualitative interviews (*n* = 27) with Head Start supervisors (*n* = 4), teachers (*n* = 7), teachers’ assistants (*n* = 4) and ancillary staff (*n* = 4) and parents (*n* = 8) to explore stressors, support mechanisms, and interest in an intervention. Findings are detailed in a separate publication (under review) however we identified that the teachers feel stressed, depressed, and struggle with multiple physical health issues (diabetes, heart problems), have experienced numerous traumatic incidences (tragic loss of family members to suicide, drug overdose or accidents), lack support for their own health and well-being and don’t seek help for their stress either due to mistrust of providers who are not Native American, time constraints or distance. They expressed interest in an intervention that would help decrease their stress and promote their psychological well-being.

To date organizational support has focused on how the Head Start teachers can better care for the children with no attention in training or services placed on addressing the health needs of the teachers. Head Start administration expressed interest having an intervention implemented that would support the psychological health of the teachers.

Individual interventions that have been implemented to help reduce stress and promote well-being in Head Start teachers include mindfulness-based cognitive therapy, access to mental health consultants and social workers, and self-care and classroom-management skills [25–27]. To our knowledge these evidence-based practices (EBPs) have not been adapted to consider the cultural values and unique challenges that may affect the well-being and levels of stress experienced by reservation-based Head Start teachers [28]. Some organizational level changes that the OHS are working to implement nationally to promote a workplace culture of support post COVID-19, include redesigning workspaces, making mental health consultants available, and providing financial incentives to reduce exposure to stressors [29]. Occupational data suggests that combining organizational and individual level approaches, especially with teacher input can be more effective than one or the other being implemented on its own [30]. It is important to acknowledge that the intervention implemented in this study is focused on individual-level psychological health behaviors.

**Fig. 1:**
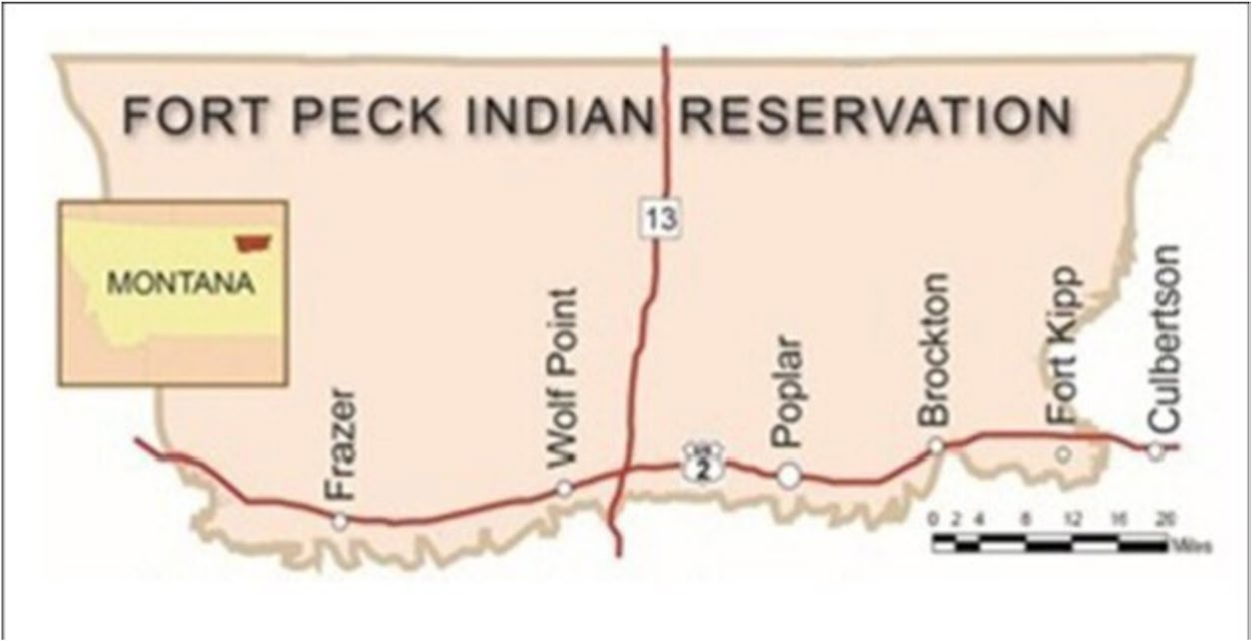
Fort Peck communities Copyright © 2022 Rink, Firemoon, Anastario, Johnson, GrowingThunder, Ricker, Peterson and Baldwin. https://creativecommons.org/licenses/by/4.0/

This article describes the implementation of a prototype 5-session intervention designed to decrease stress, and depression and increase well-being, cultural identity, and communal mastery in Fort Peck Head Start teachers. This intervention was adapted from an existing 12-session culturally informed intervention called *Little Holy One* that was originally designed for Head Start parents and children, but we felt that with adaptation it would be appropriate for Head Start teachers. On the Fort Peck reservation, there are five Head Start centers that span all districts within Fort Peck. Located in the towns of Frazer, Wolf Point, Fort Kipp and Poplar each center has its own stand-alone building. The Head Start administration office is in a separate building in Poplar and oversees all Head Start centers on the reservation. There are 21 Head Start teachers and teachers’ assistants which aligns with national Head Start regulations on teacher to child ratios allowing them to serve 300 low-income reservation-based children. Figure 1 identifies the towns where the Head Start centers are located on the reservation [31].

Here we describe the implementation of a culturally informed intervention and assess its feasibility, acceptability, and appropriateness and intervention effectiveness on decreasing stress, prompting cultural connection, and strengthening resilience and well-being. With this we hope to address the following gaps: There are few published studies assessing the feasibility of a culturally grounded treatment and to our knowledge there are no studies that have specifically studied stress in reservation-based Head Start teachers.

The primary goal was to assess feasibility and acceptability of the adapted cultural intervention and secondarily, using a mixed methods approach, to ascertain if there was any reported benefit or improvement in the well-being of intervention participants.

## Methods

### Ethics approval

The research team met with the Fort Peck Tribal Executive Board, consisting of Assiniboine and Sioux tribal leaders. Responsible for governing all matters involving tribal members and jurisdictional areas the board granted the research study a Tribal Resolution (Resolution #30-348-2020-03). This established the tribes’ rights to protect their intellectual property and Indigenous knowledge. It also provided approval for the team to apply for funding and conduct a study with Fort Peck Head Start teachers. Institutional Review Board (IRB) approval was granted by the Johns Hopkins University and the Fort Peck Tribal IRB.

### Community based participatory research

This exploratory study was embedded within a Community Based Participatory Research (CBPR) and a mixed methods social justice design that centered community needs, partnership, co-learning, and sustainability [32]. This philosophical foundation informed the research process infusing a value-based perspective into the mixed methods study [33]. It places emphasis on actively engaging participants in the research and in bringing about change for individuals and communities [32]. Table 1 outlines the steps and principals of CBPR and how we linked them to this study. The research team acknowledges the distrust that Native American communities have towards academic institutions and researchers, the result of colonization and unethical research practices inflicted on their peoples in the past [34].

**Table 1:** Steps and principles of CBPR and how they link to this research study

**Steps of CBPR** [35]	**Principles of CBPR** [36]	**CBPR Actions for this Study’s Aims**	**Strengthens the science by…** [37]
Defining, engaging community; identifying community needs and research	Recognition of the community as a unit of identity; recognize Tribal sovereignty; building on strengths and resources within community; collaborative partnerships; integration of knowledge and action for mutual benefit of all partners.	Tribal IRB; formation of Tribal Advisory Board (TAB); background research; stakeholder interviews, focus groups; building and maintaining trust; consensus of research goal and development of questions. Oversight by TAB.	Ensures Relevance
Design/hypothesis testing; responsible conduct of research	Promotion of co-learning and an empowering process that encourages social equality; cyclical and interactive process	Community relevant outcome measures; adapting modules of *Wakȟáŋyeža*, through iterative work with TAB; implement with ongoing oversight and problem solving with TAB and mentorship from Tribal Elders and Tribal Head Start admin; employing local tribal members on research team.	Enhances Rigor
Analysis; interpretation of results; dissemination; action	Focus on health from positive and ecological perspectives; Dissemination of findings and knowledge to all partners	Refinement based on new understandings; review and interpretation by all members of team; joint publications; collaborative conference presentations; presentations to community and executive board. Data owned by tribe. All manuscripts sent for approval by Tribal IRB prior to submission.	Extends Reach

The research team was able to build upon trust that had developed over a 10-year period between the Fort Peck community and a Native American researcher who helped initiate meetings and connections with tribal leaders and members. To further promote trust, reduce power imbalances inherent in academic community partnerships, and bolster community capacity we established a tribal advisory board.

Tribal advisory boards are made up of community members who share a common identity, history, language, and culture [38]. Our board consisted of a Head Start supervisor, a Head Start teacher, a Head Start parent, a cultural advisor, a public-school educator (and Head Start parent), and a Head Start grandparent. Their role was to oversee the adaptation process, pilot the pre and posttest surveys, provide the research team with culturally appropriate guidance, and communicate with the community as needed. Each board member was paid an honorarium of $100 per meeting in appreciation for their time, expertise, and cultural knowledge. By including community members and Head Start administration in the adaptation and intervention implementation, we aimed to increase the intervention’s sustainability and acceptability [22].

Finally, the research team also participated in activities not directly related to the research such as teaching yoga and helping teachers become comfortable with online formats during the COVID-19 pandemic. This helped to further build trust and relationships and develop comfort with the academic-community partnership. This partnership allowed the combination of community and researcher perspectives and expertise which facilitated understanding of the data generated. This increases confidence that the adapted intervention had the potential to fulfill a need identified by the participants and larger community.

### Conceptual model

**Fig. 2:**
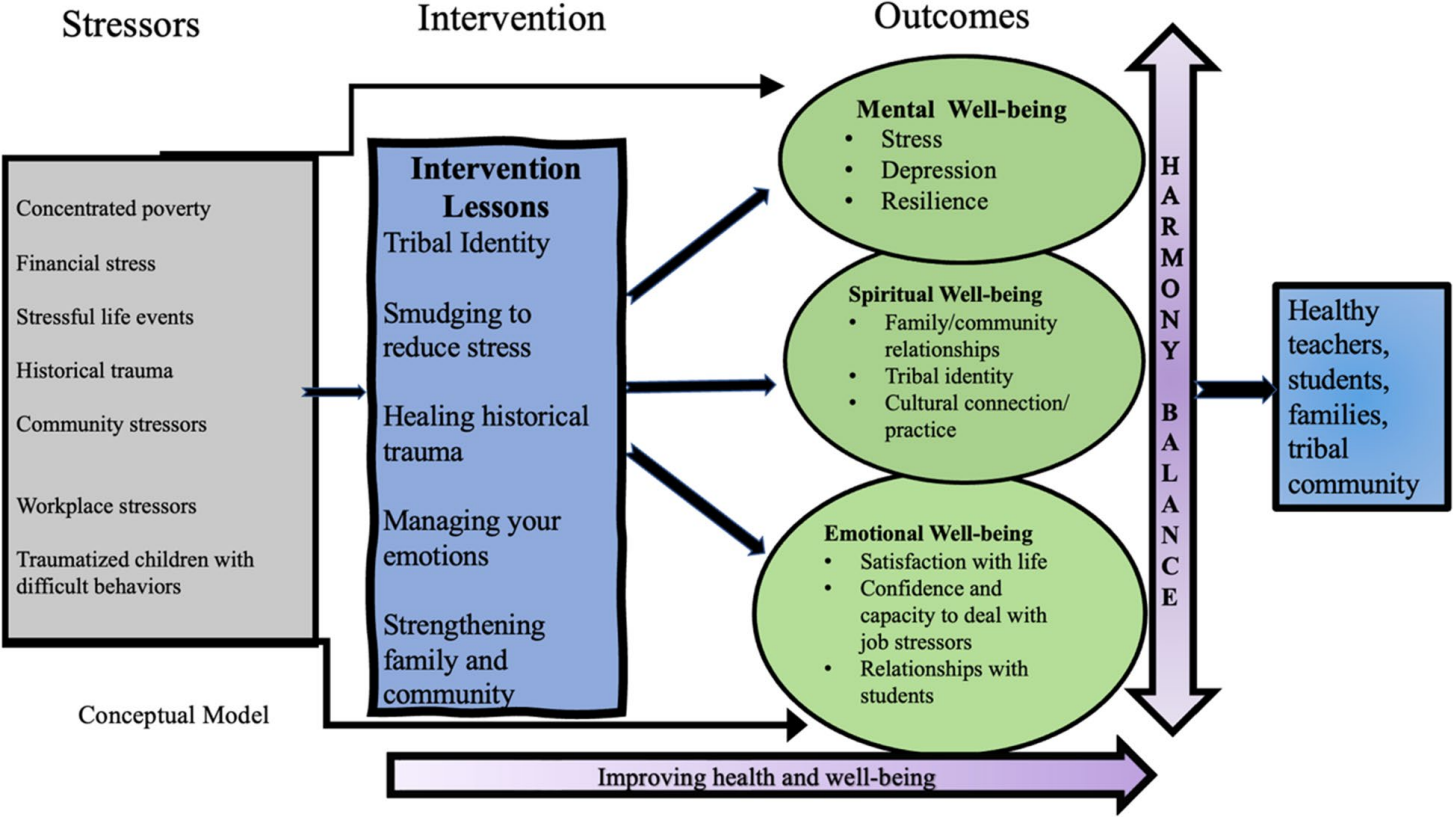
Conceptual model

To ensure the study was grounded in a strengths-based approach, it was guided by a conceptual framework informed by the Indigenist Stress-Coping model [39, 40] and the Native American Framework of Health and Well-being [41]. See Fig. 2. The conceptual model, while acknowledging the historical, contemporary, and work-related stressors and traumas likely to affect the Fort Peck Head Start teachers, places emphasis on the strength and capacity of culturally specific factors to enhance and promote teachers’ psychological, emotional, and spiritual well-being. Our goal was to provide Head Start teachers with support mechanisms and access to protective factors that could enable them to decrease their stress, cope with trauma, understand resilience, and promote well-being.

Based on our qualitative findings with the Fort Peck Head Start teachers we decided to focus on utilizing culture as a tool to promote psychological health and reduce stress and chose to adapt an existing cultural intervention called Wakȟáŋyeža (Little Holy One).

### Little Holy One

Our 5-session intervention was adapted from a larger 12-lesson curriculum called Wakȟáŋyeža, a culturally informed intervention hereafter referred to by its translation, *Little Holy One* (ClinicalTrials.gov: NCT04201184). *Little Holy One* is a randomized controlled trial (RCT) of an intergenerational intervention designed to reduce stress and trauma-related symptoms among parent-child dyads of Head Start children ages three to five [6]. This RCT is focused only on the parents and children within the Head Start program and does not involve the Head Start teachers or teachers’ assistants which was this study’s target population. *Little Holy Ones* four cultural lessons are designed to support the psychological health and well-being of parent-child dyads by (a) promoting tribal identity; (b) promoting communal mastery (group efficacy); (c) addressing contemporary and historical trauma, and: (d) promoting smudging. Although originally designed for Head Start children and their parent(s) we felt that the content could be adapted for the Fort Peck Head Start teacher context.

### Intervention adaptation

The process of adapting and developing this 5-lesson teacher intervention is described in detail in another publication [42]. Briefly we took themes from the qualitative data and used them to inform the adaptation of *Little Holy One* to be relevant for teachers. Within the CBPR framework the team adapted the intervention following a method called ADAPT-ITT [43]. ADAPT-ITT is an 8-step prescriptive process that guides researchers through intervention adaptation so that a rigorous replicable method is followed. This method was designed to be thoughtful about cultural context, sustainability, and targetable to diverse populations which helped ensure that we centered community values and capacity as we worked to adapt the *Little Holy One* lessons for Head Start teachers. Furthermore, a rigorous adaptation process helps to enhance intervention fit, improve effectiveness, and maximize fidelity during implementation [44]. With support from the principal investigator of *Little Holy One*, our tribal advisory board and Head Start administration, the research team worked with these five lessons to develop a culturally based well-being promotion and stress-reduction intervention for Head Start teachers. Table 2 outlines the key adaptions that come about through the ADAPT-ITT process.

**Table 2:** Key adaptations to the original intervention

**Adaptation**	**Rationale**	**Supporting Quotes from Adaptation Meetings**
Change format from individual delivery to group sessions	The teachers may feel more comfortable in a group format and could support each other through tough conversations.	*“We have each other’s backs.”* *“They mentor each other”* Board members
Focus on the Cultural lessons	Even though many identify as Christian there is strong connection with place and tribe	*“Some head start teachers might be Christian and not want to smudge –so it’s important to have that aspect of respect. We know that people don’t practice that way, but we respect other practices. *Board Member
Change the order of the lessons	The four cultural lessons are no longer buffered by the other eight lessons from LHO	*“We want them to be supported, end on a positive note and not stir things up at the end”* Board member *“In addressing Historical Trauma, we are bringing out the resiliency, the love. We are bringing out all that love for our family. But all that suffering we must be careful to support and focus on strengths.”* Board Member *“Provide an opportunity to smudge each lesson – the smoke connects you to creator”* Board Member
Add a session that deals directly with stress and depression	Because the qualitative data showed the teachers to be stressed and depressed the board wanted these issues directly addressed and discussed.	*“A lot of them are grandmas and aunties raising kids. Some are older still not retiring because of so many commitments, they are just used to doing it every day that they don’t even know that they are stressed.”* Board member *“In some of those homes they [teachers] don’t even have time to worry about stressing cos they are worrying how to get food, laundry soap, to keep family going.”* Board member
Delivery of cultural lessons by a Native community member	Participants are likely to be more responsive to a Native person delivering Native content	“*Natives are tired of Whites telling them about their culture. But the stress lesson should be delivered by the nurse as she is the expert”* Tribal Elder *“Some of our relatives that are drinking or addicted are suffering from something that they don’t even understand themselves.”* Board member *“All should have the cultural lessons especially. as all the children in Head Start are Native.”* Board Member
Deliver sessions at each center	Each center has its own culture and will reduce participant burden	*“These lessons you are encouraging them to talk together, and that is important.”* Board member

During the adaptation process for the current effort, [42], a fifth lesson from the *Little Holy One* curriculum was added to the planned intervention. The fifth lesson, Managing Your Emotions, is from the Common Elements Treatment Approach (CETA) and focuses on recognizing and dealing with depression, anger, and stress. CETA is an EBP designed to reduce mental health problems in low- and middle-income countries and can be delivered by community-based paraprofessionals [45]. Through listening to the advisory board, teachers, and the larger community we learned, it was important to add specific education about stress and depression, how it manifests and how to manage it. During one adaptation meeting a board member asked if the cultural lessons would deal directly with stress given that the qualitative data pointed to the teachers being unaware of or not dealing with their stress. The *Little Holy One* community research assistant proposed adding a lesson from the other *Little Holy One* eight lessons called “Managing Your Emotions”. This module from CETA focuses on managing stress and depression and was deemed important to the success of the intervention by directly addressing workplace stress [45]. The *Little Holy One* research assistant delivered the lesson, and the board suggested a Native visualization to replace the modules meditation script. Otherwise, they felt that the module format of working through recognizing stress, identifying causes of stress and how to manage stress would promote fruitful discussion among participants and help them to identify and deal with stress triggers in their lives. Table 3 describes the five lessons included in the intervention.

**Table 3:** Description of the five lessons included in this intervention

Cultural sessions	Description of Sessions	Session activities and time to complete
Promoting Tribal Identity	Connects one to the Creator, responsibility to live a good life by walking spiritual path	Practice greeting of relatives in Nakoda and Dakota. Traditional naming 1 h
Smudging	Therapeutic healing practice to resolve unsettling feelings and thoughts	Smudging together; smudging as a daily routine 45 min
Healing Historical & Contemporary Trauma	Identify imbalances in physical, emotional, mental, and spiritual domains created by historical trauma.	Identifying and coping with effect of historical traumas. Strength and resilience Forgiveness exercise Smudge at end 1 h
Strengthening Family and Community Cultural lesson	Therapeutic value of connectedness to relatives and community	Knowing our relatives Family tree exercise. My friends and family exercise 1 h
Common Elements Treatment Approach (CETA)		
Understanding our Emotions	Understand association among thoughts, feelings, and behavior	Identifying depression, managing anger and stress; working through challenges Visualization activity 1 h

### Participants and recruitment

Head Start teachers and teacher assistants were informed about the study during a reservation wide Head Start staff meeting, and flyers describing the study were hung at each center. Follow up emails were sent three times from the Head Start Supervisor with teachers contacting her rather than the researchers to express interest. A follow up in-person visit 2 weeks before the start of the study by researchers to each Head Start center to discuss the study and answer any questions was the most successful strategy as this was when those that expressed interest and others committed to participating in the study. Final recruitment included 18 participants. Eligibility criteria were that participants needed to identify as Native American, currently work as a Fort Peck Head Start teacher or teacher assistant, be 18 or older, and speak English.

The 5-session intervention was implemented in April and May 2022 over a 7-week period. Participants were compensated with a $35 gift card for each lesson attended. An extra $35 gift card and entry into a raffle to win a prize were given to those who completed all five lessons. Written consent was obtained prior to the start of the first session. Several participants indicated that they did not want sessions or focus groups to be recorded, therefore observations and notes were taken by a research team member throughout the study. The four cultural sessions (Tribal Identity, Smudging to Reduce Stress, Healing Historical Trauma, Strengthening Family and Community) were facilitated by a community paraprofessional from Fort Peck while the CETA lesson, Managing Your Emotions, was implemented by the first author who is a nurse.

To reduce burden on participants, the intervention sessions were conducted at each Head Start center (Frazer on Mondays, Wolf Point on Thursdays, Poplar, and Fort Kipp on Fridays).Head Start administration allowed time during works hours for implementation of the sessions. The Tribal Handbook stipulates that teachers can use 30 min of paid time a day for health e.g., go for a walk and the Head Start administration felt it could be utilized for a scheduled weekly session to support mental health. While this was an intense weekly schedule for the researcher and facilitator the goal was to support retention throughout the whole study. Sessions were conducted in private Head Start meeting rooms at each center to allow for confidential conversation. Food was provided. Because many participants were parents or grandparents, children were allowed to come and go with snacks and activities provided so their safety was ensured while their caregivers were busy with the intervention.

### Facilitator trainings

Three facilitators were hired from the community to implement the adapted intervention. All were Native American, female, aged in their thirties and resided on the reservation. They had all been trained as facilitators for the *Little Holy One* study and so were familiar with original module content. Two training sessions were conducted where the research team described the module adaptations and the rationales. Then the facilitators practiced implementation of each adapted module with the research team and other facilitators role playing as participants. One of the original designers of the *Little Holy One* cultural modules joined via Zoom and checked for fidelity to the core components of each module. As a group the team decided that facilitators would be assigned to a center and deliver all modules over the study period. This was preferred by the facilitators to the other option of “specializing” in one or two of the modules and delivering them to each of the centers. It was felt that it would be better to develop relationships with ones assigned center and that this might facilitate participants becoming more relaxed in the study environment and therefore participate and gain greater benefit from the sessions. The exception was the CETA module which was to be delivered by one of the academic research team. She would deliver that module to each of the centers.

Just prior to commencing the study one facilitator accepted a position on Tribal Council which mandated that she could not work on any other job and a second facilitator decided that she was not confident enough to deliver the material and run the sessions. This left one facilitator and one research team member to deliver all the sessions. There was no time to find, hire and train a new facilitator. To make the schedule more manageable we decided to join the Fort Kipp Head Start teachers with the Poplar Head Start teachers via Zoom. The first author traveled to Fort Kipp each Friday and remotely joined the Fort Kipp teachers to the session. The facilitator was paid $25 per session and brought tremendous expertise to the job. Her MPH qualification, strong cultural training, being stepped in the cultural practices in her personal life including being a competent Nakoda speaker gave her confidence in the delivery of the cultural lessons and a commitment to the success of the project.

### Research design and analysis

To examine feasibility, acceptability, and appropriateness of the intervention, we used an embedded explanatory mixed methods design in which qualitative interviews with participants and facilitators were used to help explain survey and questionnaire results.

### Pretest/posttest survey

Paper and pencil pre and posttest surveys were administered prior to the start of the first session and upon completion of the last session, respectively. Paired T-tests and Hedges G effect sizes were calculated to assess effect and direction of change in the outcome measures. Based on our conceptual model, our theory was that the four cultural lessons and one CETA lesson would result in the following self-reported outcomes: a decrease in perceived stress; a decrease in depression; and an increase in satisfaction with life, tribal identity, communal mastery and resilience.

Careful attention was taken to use measures validated in Native American or Indigenous populations. A pilot of the pretest/posttest survey with instructions and measures was conducted with our tribal advisory board to assess acceptability and appropriateness. The board discussed order of the measures and clarity of instructions. Minor modifications were made based on the board’s feedback and all the following validated measures were accepted for inclusion: The *Satisfaction with Life Scale* [46] was used as a measure of well-being based on its success in other studies of subjective well-being in Indigenous populations [47]. It consists of 5 items on a 7-point Likert scale with a Cronbach’s alpha of 0.78.

The *Perceived Stress Scale* 10 (PSS-10) [48] was used a measure of stress. It is widely used and has been successfully used in Native American populations. It consists of 10 items on a 5-point Likert scale with a Cronbach’s alpha of 0.78. Summed scores above 20 are reported as “high stress” while scores from 13–19 are considered “average stress.”

The *Center for Epidemiologic Studies Depression Scale Revised* (CESD-R-10) [49] was used to measure depression. The CESD-R is a self-report measure consisting of 10 items based on the Diagnostic and Statistical Manual of Mental Disorders (DSM-IV) diagnostic criteria for major depression. It has a Cronbach’s alpha of 0.86. Scores range from 0–30 with scores higher than 10 indicating depression. This measure has been validated in Native American populations [50].

The *Communal Mastery Scale* [51] was used as a measure of self-efficacy. It was developed specifically for Native American contexts and uses two commonly employed measures of mastery and self-efficacy. It contains 10 items on a 4-point Likert type scale with a Cronbach’s alpha of 0.85.

The *Orthogonal Cultural Identification Scale* [52] was used to measure tribal identity. It was modified to be specific to the Assiniboine and Sioux tribes and was used in a previous study on the Fort Peck Reservation [6]. It contains six items with a Cronbach’s alpha of 0.90.

The *Connor Davidsons 10 item Resilience Scale* (CD-RISC-10) [53] was used to measure resilience. This scale has been found to more effective with Native American populations than the Connor Davidsons 25 item Resilience Scale CD-RISC-25 [54]. It consists of 10 items of a 5-point Likert scale with a Cronbach’s alpha of 0.85.

The *Benevolent Childhood Experiences Scale (BCE)* was used to measure childhood resilience [55]. It has been used successfully with Native American populations [56] and consists of 10 items using a yes/no scale in adults who have experienced adversity.

The *Adverse Childhood Experiences Measure* (ACES) was used to measure childhood adversity. It has been validated in many contexts and is a sum of different types of abuse, neglect, and other family stressors experienced before age 18. It consists of 10 yes/no items with a Cronbach’s alpha 0.67.

The *Stressful Life Events Questionnaire* (SLEQ) [57] was used to contextualize the historical traumas and inequities suffered by Native American peoples and to capture potential differences in exposure with this population [58]. This self-report measure assesses life-time exposure to traumatic events and has been shown to more accurately capture traumas experienced by Native American populations than the ACE score [6, 58]. This questionnaire consists of 13 yes/no items with space for with free text responses. It has a Cronbach’s alpha of 0.73.

The *Historical Loss Scale* [59] uses 12-items to indicate the frequency that Native Americans think about loss of their culture, land, and peoples due to colonization. It has a Cronbach’s alpha of 0.93. The *Historical Trauma Checklist*, developed during focus groups on the Fort Peck reservation, contains 15 items asking how often a person thinks about different historical trauma experiences [60].

Analysis of the surveys was conducted in STATA [61], where data was cleaned and assessed for missingness. Participants who did not fill out sections in the pretest or posttest were removed from analysis of that specific outcome.

### Qualitative data collection

The first author attended each session with the facilitator to provide practical support and collect observational data. We decided to keep the same person doing the observation to provide consistency as having a different member of the research team attending different sessions could potentially make the teaches feel self-conscious or unsafe. Moreover, the first author was to implement the CETA module and would thus become a familiar face to participants before leading a session. Notes from the sessions and post-session debriefs were typed up after each session so that they could be presented to the full team at the end of the intervention. The facilitator and observer role were supposed to be switched during the CETA module, but the facilitator fell ill that week and so the first author took notes from memory after delivering each session that week.

Upon completion of the study, after participants completed the posttest survey, we conducted focus groups to ask participants about their experience of the intervention using the following prompts: did you notice any effect from the intervention? What other outcomes would you like to see? Was the intervention acceptable to you? For the survey, we asked whether there was any discomfort filling it out, was the length appropriate, were any questions difficult to answer, was there any confusion about the instructions, and if the instructions guided participants through the survey. Due to unexpected circumstances (the sudden tragic death of one of the facilitator's family members which necessitated delaying the study) during study implementation, the final session and focus groups had to be conducted combining groups together. This was not ideal as each center had its own culture and relationship with the facilitator. Some teachers were more reluctant to speak in the larger group and thus we did not get as much verbal feedback as we had hoped. Two 30-min focus groups were conducted consisting of eight participants and ten participants each. The focus groups were led by the first author with notes taken by the facilitator which were then complied and discussed with the team and Head Start administration during a postintervention debrief.

### Fidelity

To measure intervention fidelity, the first author attended all sessions to collect data and observe interactions, examine intervention attendance or early departure, and note number of interruptions and attention span. Facilitator adherence to lesson delivery was first tested with one of the developers of the *Little Holy One* intervention prior to the study commencing. During intervention implementation, the first author used a quality assurance form for each session that noted duration of session, time spent on warm up, reviewing of previous session, adherence to the prescribed order of activities, doing the activity together, provision of handouts and scheduling next session. They also noted the facilitator’s relationship with participants, looking for a professional but positive working relationship and sensitivity to culture and religious differences. Specifically, the quality assurance form measures three areas, (a) Lesson Structure and Talks-10 items, using scores ranging from 1–4 with 1 being (none) to 4 (exceeds expectations), (b) Relationship – six items using same scoring of 1–4 and (c) Adherence, Competence, and Flexibility with six items each scoring 1–4. The first author also gathered qualitative data by noting comments by participants throughout each lesson and conducting a debrief with the facilitator after each session.

### Acceptability, appropriateness, feasibility

To measure intervention acceptability, appropriateness, and feasibility, we administered the Acceptability of Intervention Measure (AIM), Intervention Appropriateness Measure (IAM) and Feasibility of Intervention Measure (FIM) [62] at the completion of the intervention. These measures are viewed as leading indicators of implementation success and provided an opportunity for participants to retrospectively assess their perspective of the program. Each measure uses a 5-point Likert scale using scores ranging from 1(completely disagree) to 5 (completely agree) and all have demonstrated structural and discriminant content validity and test–retest reliability [62]. AIM with four items asked participants if the cultural lessons met their approval, were appealing and whether they liked and welcomed the intervention. IAM with four items asked participants to rate if the cultural intervention was fitting, suitable, applicable and if it seemed like a good match. FIM with three items asked if practicing activities learned such as smudging seemed possible, implementable, and doable.

Qualitatively, study participants and Head Start administration were asked what they found acceptable or not about the intervention and whether attending sessions long-term (1–3 years) was appropriate and feasible.

## Results

### Quantitative outcomes

Table 4 outlines participant demographics. Of the 18 study participants, 87% were over 50 years of age and 65% had been teaching at Head Start for more than 10 years and all identified as Native American

**Table 4:** Demographic data

**Gender**	**N (18)**	**Valid %**
Female	18	100
**Age**		
31–40	2	13
41–50	0	0
51–60	4	27
61–70	5	33
71–80	4	27
**Tribal Affiliation**		
Assiniboine	5	29
Sioux	4	24
Assiniboine & Sioux	6	35
Other	2	12
**Marital status**		
Single	15	83
Married	3	17
**Education**		
High school	6	33
Associates	1	6
Bachelors	11	61
Masters	0	0
**Household Income**		
< 20,000	5	29
20–40,000	9	53
40–60,000	3	18
**Years Teaching at Head Start**		
2–5 years	2	11
5–10 years	4	24
10–15 years	1	6
15–20 years	4	24
> 20 years	6	35

Table 5 details the findings for all the pretest screening measures.

**Table 5:** Pretest screening measures

Measure	Most frequently chosen options per measure	Percentage of participants
Adverse Childhood Experience (ACEs) 10 items Overall mean score: 3.4	Did you live with anyone who was a problem drinker or alcoholic or who used street drugs?	61%
	Was a biological parent ever lost to you through divorce, abandonment, or other reason?	44%
	Did a parent or other adult in the household often or very often… swear at you, insult you, put you down, or humiliate you? or act in a way that made you afraid that you might be physically hurt?	44%
	Did a parent or other adult in the household often or very often… push, grab, slap, or throw something at you? or ever hit you so hard that you had marks or were injured?	44%
Stressful Life Events Questionnaire 13 items	Immediate family member, romantic partner, or very close friend died because of accident, homicide, or suicide.	61%
	Being ridiculed, put down or ignored by a parent, romantic partner, or family member.	55%
	Reported that as an adult they were kicked, beaten, slapped around or otherwise physically harmed by a romantic partner, family member, stranger, or someone else with some reporting injuries such as broken ribs and jaws.	44%
	Being physically forced to have intercourse, or oral or anal sex, against their wishes, or when they were helpless such as being asleep or intoxicated.	44%
Benevolent Childhood Experiences (BCE’s) Scale 10 items Overall mean score 8.6	Growing up I had:	
	At least one caregiver with whom you felt safe.	100%
	At least one good friend	100%
	Beliefs that gave you comfort	100%
Historical Loss Scale 12 items	Losses that you have thought about daily:	
	The losses from the effects of alcoholism on our people	33%
	The loss of respect by our children and grandchildren for elders	33%
	The loss of our people through early death	27%
Historical Trauma Checklist 15 items	Which of the following have you or your family experienced?	
	Grandparents, great-grandparents, or elders attended mandatory boarding school: Did they…	61%
	Experience loss of naming or a forced English name	61%
	Experience separation from family members	61%
	Loss of language	55%
	Hair being cut	55%

**Fig. 3:**
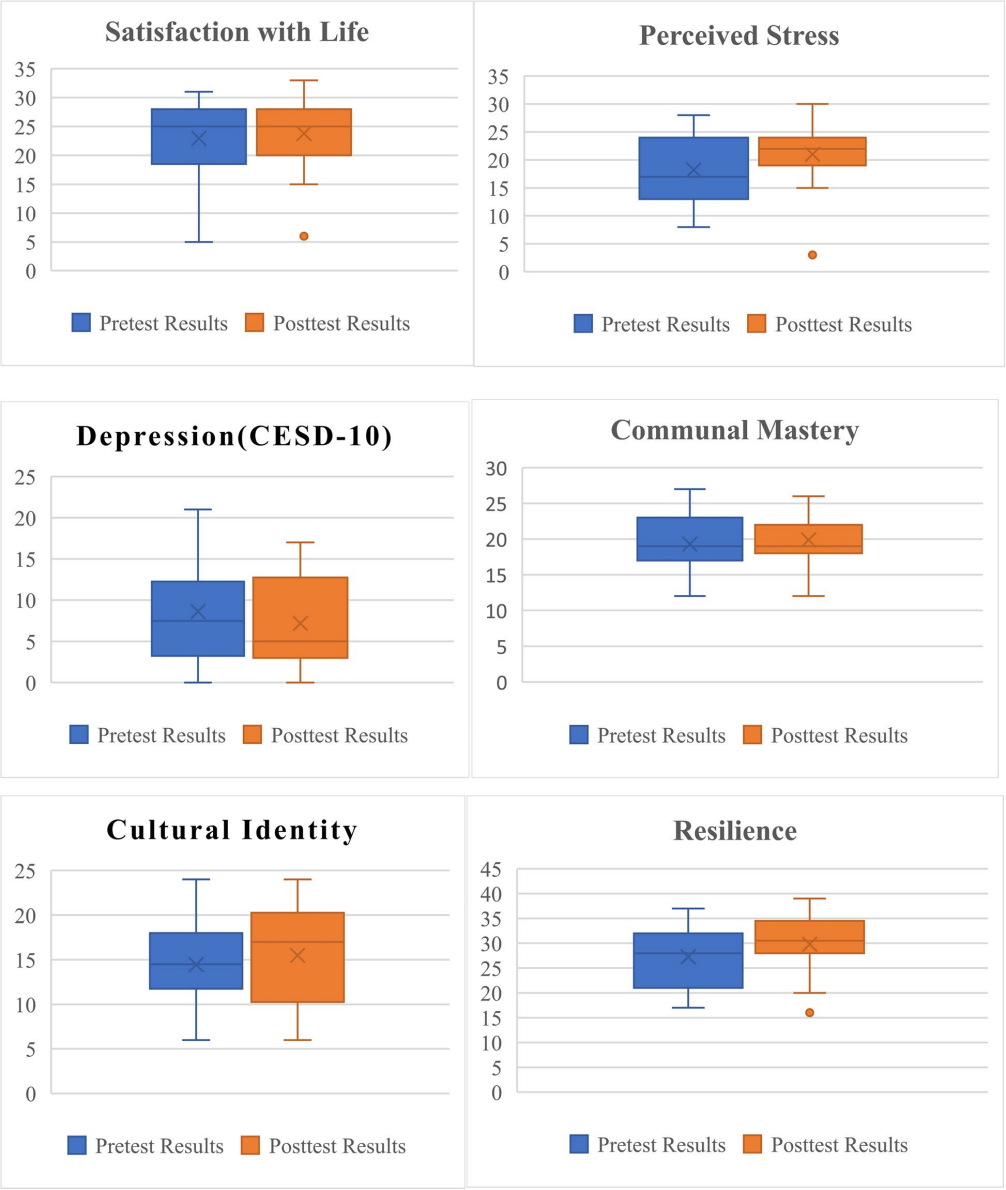
Boxplots of the pretest posttest means

Table 6 outlines the means, confidence intervals and Hedges G for outcome measures that were tested pre and post intervention. While we conduced paired t-tests we were using effect size rather than p-value to evaluate meaningful change since this was a feasibility study with small sample size. Figure 3 depicts the same results in graphic form.

**Table 6:** Outcome measures for pretest posttest survey, means, standard deviation and Hedges g

**Measure**	**N**^**a**^	**Pretest** **Mean (SD)**	**Posttest** **Mean (SD)**	**Difference** **Mean**^**b**^** (SD)**	**t-stat**	***P*-value**	**Effect Size**^**c**^** (CI)**
Satisfaction with Life Scale	16	24.1 (5.7)	24.9 (5.3)	-.81 (4.4)	-0.73	.47	.14 (-.53,.82)
Perceived Stress Scale	14	18.3 (6.1)	20.8 (6.5)	-2.5^b^ (7.01)	-1.33	.14	.39 (-.35,1.11)
Depression. CESD-10	16	8.6 (6.2)	7.2 (5.4)	-1.5 (5.9)	-.97	.35	-.24 (-.92,.22)
Communal Mastery Scale	17	19.3 (3.8)	19.9 (3.4)	1.1 (3.9)	-.60	.55	.16 (-.50,.81)
Tribal(cultural) Identity Scale	18	14.4 (4.6)	15.5 (5.6)	1.06 (5.3)	.85	.41	.21 (-.44,.84)
Resilience. CD-RISC-10	17	27.6 (6.2)	28.8 (7.3)	-1.8 (8.4)	.057	.57	.24 (-.41,.88)

^a^Analyses only included participants who completed both pretest and posttest surveys

^b^Negative values indicate decreases compared to baseline

^c^Effect size constitute Hedges g: small effect = 0.2, medium effect 0.5, large effect 0.8

We hypothesized an increase in satisfaction with life*,* but it did not meet the threshold for a small effect size. For self-perceived stress we hypothesized a decrease in stress, however the effect size demonstrates a small to medium positive effect size indicating that contrary to our hypothesis the teachers indicated an increase in stress. However, with depression there was a small but meaningful negative mean difference score in line with our hypothesis that there would be a decrease in depression. Furthermore, looking at individual data, six participants scored over 10 in the pretest survey with a score equal to or above 10 considered depressed. On the posttest survey four of these participants had scores lower than 10. For tribal identity and resilience there were small but meaningful positive effects while communal mastery did not meet the threshold for a small effect size when we hypothesized that there would be an increase.

### Qualitative data

Observations during each session showed teachers participating in the activities which stimulated conversations about their experiences of trauma, support, and coping mechanisms. When presenting the cultural lessons, the facilitator worked to elicit teachers’ experience and understanding of traditional stories and guided conversations from those who have little or no experience with the traditions and stories. The facilitator and group participants created a supportive environment as difficult conversations and emotions were expressed, with some participants stating that they had never spoken to anyone about some of the incidents discussed. There was also a lot of laughter and teasing of each other and the researcher as participants worked through activities together. Table 7 outlines the observational and qualitative quotes integrating them with the quantitative findings.

**Table 7:** Joint display integrating quantitative and qualitative results

**Quantitative Findings**	**Observational Data**	**Participant Quotes within Sessions**	**Post-Intervention Focus Groups**
**Stress**. Small to medium positive effect size	The “Forgiveness brings Healing” activity from the Healing Historical Trauma session: prompted teachers to speak about traumas that have deeply affected them and their relationships to authorities or others in the community. Two participants revealed they had never spoken about these incidents to anyone before.	*“I have anxiety triggers that I am just now paying attention to. Sometimes when I am mad or crappy its cos of a past trauma.”* (Participant Healing Historical Trauma session)	“*It was helpful – made you feel you could go another week”* (Participant FG1) *“Just talking helped out. It made me feel good going home”* (Participant FG1) “*Thank you. All these traumas I just thought they were part of my life. But [during the intervention] I got to know myself and how they still affect me. I haven’t looked at it like that before.”* (Participant 3 months post intervention)
**Depression** Small but meaningful negative mean difference score	Three participants spoke about family loss to drugs and the constant fear that they live with that they will get another call saying another child or grandchild had died of an overdose.	*“I just know that when I lost my husband, I needed a lot of support, but I didn’t have it.”* (Participant CETA lesson)	
**Communal Mastery**. Did not meet the threshold for a small effect size.	Family tree exercise for the Strengthening Family and Community session elicited animated conversations about relatives, how people are connected, revealing unknown genealogical connections as well as a discussion with one group about the origins of the Assiniboine and Sioux being placed on the reservation and the effects that this still has on community dynamics.	*“How lonely it was growing up as I didn’t have family until I moved back here. I feel at home here, safe.”* (Participant, Strengthening Family and Community Session) *“As hard as COVID was I didn’t want to be anywhere else but here. Every time in quarantine there was stuff left at the door.”* (Participant, Strengthening Family and Community session) *“Innate in us is to think about each other even in times of hardship.”* (Participant, Strengthening Family and Community session)	
**Tribal Identity** small but meaningful positive effects	Extensive discussion in two groups about boarding schools: *I didn’t ask my mom about those boarding schools. She never talked about it negative or positive. Weren’t allowed to speak language cos she was taught it was wrong.* (Participant, Healing Historical Trauma session) *My mother talked about it – don’t look in a white man’s eyes as they are evil. But my mother would look them right in the eye so that they would know she was strong.* (Participant, Healing Historical Trauma session) Traditional Naming activity: animated participation “*It’s really important when teaching these lessons to affirm why our families may or may not have continued these traditions and that it’s ok to not know certain things or do certain things. It’s about normalizing our cultural practices, beliefs, and understandings.”* (Facilitator in post-session de-brief)	*“Helps with a place that you can call home when you have a traditional name.”* (Participant Promoting Tribal Identity session)	
**Resilience** Small but meaningful positive effect	*“Our whole community/nations are still there … despite the damage.”* (Participant, Tribal Identity session)	“*It was hard for me. I just, I don’t do drugs, and I don’t drink. I know when my husband died, I drank, and then I just put myself in treatment because I knew I had to go to treatment.”* (Participant, CETA lesson) “*All teachers can take a lot. All teachers can manage a whole lot.”* (Participant, Strengthening Family and Community session)	
**Timing of the Intervention** Delayed due to unexpected circumstances resulting in final session falling on the last week of school before summer break.	Observation Notes: In the last session it was noticeable that the more vocal teachers were quieter than usual. Less animation in the sessions.	*“I’m tired, just done for the year.”* (Participant, Strengthening Family and Community) when asked why she was unusually quiet in the session.	*“We are used to nothing going according to plan. It is a complicated life here on the rez.”* (Participant FG 2)
**Sustainability** Tribal handbook assigns 30 min a day for staff health. Overall attendance rate 93%	Each center had its own unique culture and relationships. While burdensome for facilitator holding sessions at each center it supported retention and potentially safety of participants to speak.	*“I look forward to it – the sessions weren’t stressful.”* (Participant, FG 1) “*Our administrator did good lettings us do this; we don’t usually get such time for us.”* (Participant FG1)	“*Visiting together we got to know each other more, know ourselves, gave more support to each other.* (Participant FG2) *“We need this support - I don’t mean a mental health counselor but if we could meet together and talk and share and support each other it would help us a lot.”* (Participant, 3 months post intervention)

Upon completion of the intervention we met with Head Start supervisors and administration. They reported that teachers had been informing them throughout the intervention how much they appreciated the sessions and expressed appreciation of the flexibility of the research team to work with each centers needs when scheduling sessions.

### Fidelity

Our goal of 12–15 participants was exceeded with a cohort of 18 participants joining the study. Participants stated that the purpose of the study was clear. The reason for travelling to each Head Start site to implement the study was to minimize teacher burden, therefore we expected high attendance rates. We also hoped that with the knowledge that the intervention had been developed with their input from the qualitative interviews and that using cultural lessons originally developed in collaboration with Fort Peck tribal elders would also support a high attendance rate. There was no loss of participants throughout the 7 weeks and attendance rate overall was 93% (there were three incidents where a participant left a session early). Thus, our recruitment strategy was successful.

Teachers reported that having the intervention held at each center made it easy for them to attend every week: “*Our administrator did good lettings us do this, we don’t usually get such time for us.”* (participant) Attention span was good, and we kept each session to an hour. Each group had its own personality and dynamics and the facilitator worked with quieter individuals to include them in the discussions.

Time to complete the paper and pencil pretest-survey survey was between 35–45 min. In the piloting of the survey with the tribal advisory board it took 30 min. Teachers reported that the instructions were clear, and they had no issues with the order of the questions. One participant noted on the survey that she was shocked by the questions about sexual abuse but still filled out that section. The posttest survey took 30–40 min to complete with participants reporting that it was not a problem to complete.

The facilitator scored high on all the quality assurance forms delivering lesson content as planned, demonstrating good knowledge of the materials and an ability to facilitate difficult or emotional conversations. Specifically, the facilitator scored 38–40/40 for each session on Lesson Structure and Talks, 15–16/16 for Relationship and 19–20/20 for Adherence, Competence and Flexibility. It was clear that having a Native facilitator from the community engendered a trusting working relationship with the teachers throughout intervention implementation as evidenced by animated participation during sessions and participants from each center expressing appreciation to the facilitator for her time and care at the completion of the final sessions: *“Thank you, you made me laugh even during tough conversations.”* (Participant Focus Group 1).

### Acceptability, appropriateness and feasibility

Sixteen out of 18 participants filled out the AIM/IAM/FIM form. Two study participants were not present. This provided data on participant perspective of implementation outcomes rather than service/treatment effects. The total mean score and standard deviation (SD) for acceptability which measures whether the intervention was agreeable was 4.25 (0.84) out of a total score of 5 with participants choosing completely agree, agree, neither agree nor disagree. Zero participants chose disagree or strongly disagree. The total mean score for appropriateness which measures the perceived relevance and fit of the intervention was 4.18 (0.86) out of a total score of 5 with participants choosing completely agree, agree, neither agree nor disagree. No-one chose to disagree or strongly disagree. For feasibility, the extent to which things learned during the intervention could be carried on by teachers scored a total mean of 4.06 (0.96). Specifically with the question “practicing smudging seems doable” three participants chose to disagree, and one completely disagree. Table 8 details each question, mean and SD for all AIM/IAM/FIM measures.

**Table 8:** Mean and standard deviations for the AIM, IAM, FIM questionnaire

**AIM, IAM, FIM**	**Mean (SD)**	**Range (1–5)**
Acceptability of Intervention (AIM)		
Total Mean Score	4.25 (.84)	
The cultural intervention met my approval	4.25 (.86)	3–5
The cultural intervention was appealing to me	4.25 (.86)	3–5
I liked the intervention	4.25 (.86)	3–5
I welcome the intervention	4.25 (.86)	3–5
Intervention Appropriateness (IAM)		
Total Mean Score	4.18 (.86)	
The cultural intervention seemed fitting	4.25 (.86)	3–5
The cultural intervention seemed suitable	4.19 (.84)	3–5
The intervention seemed applicable	4.13 (.89)	3–5
The intervention seemed like a good match	4.20 (.86)	3–5
Feasibility of Interventions (FIM)		
Total Mean Score	4.06 (.96)	
Practicing things I learned seems possible	4.20 (.84)	3–5
Practicing the cultural interventions seems implementable	4.25 (.86)	3–5
Practicing smudging seems doable	3.75 (.14)	1–5

We then met with the Head Start supervisor and administrator who conveyed that the intervention went well and that continuing some form of support for teachers was not only feasible but essential. They scheduled a meeting with the tribal council where we presented study results and submitted a request for funding to implement strategies that support teacher psychological health on an ongoing basis. Given the positive feedback from the teachers there was discussion about using the paid time stipulated in the Tribal Handbook that allows teachers to take 30 min a day for physical health (e.g., go for a walk) to be utilized for a scheduled weekly session to support mental health. Currently Head Start administration is writing a grant supported by these study results to apply for funding to implement support activities for the teachers.

## Discussion

In response to a recognized need to support Fort Peck Head Start teachers to better manage the stressors they encounter in their day-to-day work serving the developmental needs of preschool aged children and families, we adapted a culturally informed intervention designed to decrease teacher stress and promote well-being.

### Baseline measures

As we could not find any documented data on the Fort Peck Head start teachers, we included some pretest measures to gather some baseline information. The overall ACE mean score was 3.4 and research suggests that an ACE score above three especially with associated medical conditions places individuals at high risk for toxic stress and they should receive education on stress and protective buffering factors [63]. The data that was collected using the stressful life events questionnaire indicates stressors that cannot not be captured using the ACE score alone, further supporting the need to adapt ACE measuring tools to include other adversary categories that would more accurately capture the ACE score of Indigenous populations [64]. As we expected those participants that scored low on ACE measures scored very high on BCEs, but we also found that of the seven participants that had an ACE score of four or greater five of them also had a BCE score of 8 or higher. This seems to help support evidence that social support (a component of BCE’s) can buffer individuals from the effects of childhood adversity [65]. It would be interesting to explore the more nuanced view of social support in Indigenous communities e.g., connection to culture, place, and community as a buffer to the adversities of historical trauma, discrimination, and domestic violence. It was striking the percentage of participants that have experienced some form of domestic (44%) or sexual (44%) violence and the loss of someone close to them from an accident or suicide (66%). Evidence shows that women who have experienced such issues can benefit from groups where they can share and support each other [66]. Thus, inadvertently by adapting the intervention from an individual to group format we may have provided support to those who were struggling with or have survived domestic violence.

### Pretest posttest measures

Effectiveness of the intervention at the participant level was measured quantitatively via a pretest–posttest survey and qualitatively via focus group interviews to assess participant intervention experience.

#### Satisfaction with life

Post-intervention, there was no discernable shift in Satisfaction with Life. Perhaps the number of stressors and the length of time that teachers have been experiencing them requires a more sustained intervention over time to produce a significant change in such a measure. The Satisfaction with Life measure is designed to elicit participants’ global assessment of life-time satisfaction however, previous research shows that current mood and situational factors at the time of completing this measure can affect results [67].

#### An increase in stress

Results overall were positive, with the exception that one of the targeted outcomes (self-perceived stress) worsened. The reporting of higher self-perceived stress following the intervention could have been influenced by contextual factors. For example, the final session fell on the last week of school when teachers described being tired and ready to be done for the year. Teachers had also just been informed that their classroom sizes would be returning to normal, after being reduced during the COVID-19 pandemic. Teachers expressed anxiety and concern about this return to full sized classrooms. Factors such as these may have influenced participants self-perceived stress independent of the intervention. It is also possible that by educating the teachers about stress and providing knowledge and tools to manage it, their awareness of stress may have increased. This seems supported by the qualitative data quoted above, as well as other studies that found educating teachers about stress can increase their awareness of how stress is impacting them [28, 68].

At the participant level our intervention focused on modifying each teacher’s relationship to stress. Literature shows that such interventions typically show only small to medium effect sizes [67, 69]. Existing data suggests that a preventative systems-level intervention approach would be more effective than individual-level interventions at reducing teacher stress [69]. Our qualitative data suggested that many of the stressors’ teachers experience are complex and intertwined with community issues – such as drug and alcohol misuse, discrimination and historical traumas that have affected families and communities and were important to highlight and discuss. Thus, while there are no large effect sizes based on quantitative data, the small shifts in conjunction with teachers’ high engagement during sessions and descriptions of positive effects reported in post-intervention focus groups, are positive indicators of benefit. Therefore, we believe that such interventions are worthy of further exploration as means to promote teacher well-being in early childhood settings. Sources of stress at the workplace level that were identified during sessions were communicated to administration with discussions for organizational-level changes such as new classrooms and adult furniture. A multi-level approach to the well-being of these teachers is essential if a reduction in teacher stress and improvement in well-being is to be effective and comprehensive. It was interesting to note that ancillary staff at each Head Start center expressed interest in joining the study. While this was not possible it has prompted discussion about including all interested Head Start staff at centers as part of a clustered randomized trial in the future. This would help increase sample size. Including all staff at each center may also help identify other organizational-level changes that could positively affect all staff and not just the teachers.

#### Depression

Depression had a small negative effect size. This may indicate that receiving active support can buffer an individual from daily and lifetime stressors. This assumption is supported by our qualitative data. It was interesting to note that individuals who scored higher than 10 on the CESD-10, a score that indicates they are depressed, showed significant decreases in their score from pre- to post-intervention. In another study with Head Start teachers who were provided mental health support, teachers experiencing the highest levels of stress were the ones who took advantage of the interventions offered [70] – suggesting, perhaps, greater motivation among those experiencing the most distress to take advantage of intervention offerings.

#### Cultural strengths and healing historical trauma

The intervention’s emphasis on building cultural strengths and healing historical trauma was not specifically referenced by participants but there were positive shifts in communal mastery, and tribal identity. It may be that by bringing a conscious focus to these topics and encouraging participants to describe their experiences, participants became more aware of their cultural and community identity and connections [71].

#### Resilience

Resilience showed a small positive effect size. It is plausible that the intervention’s focus on culture i.e., with its focus on strengths and traditions that have survived atrocities and government policies – facilitated participants’ attunement with their psychological resilience and perseverance despite ongoing hardships [72]. This finding suggests that counselors who provide services to Native American individuals and communities should be aware of both the complex trauma that Native people have experienced, as well as the strength and agency they possess [73, 74].

### Implementation outcomes

Implementation outcomes including intervention acceptability, appropriateness, feasibility, fidelity, and sustainability were assessed using the quality assurance form, and the AIM/IAM/FIM measure, qualitative observations, and focus groups and meetings with Head Start administrative staff. The intervention was implemented with a high degree of fidelity and both quantitative and qualitative data indicate that participants found the intervention to be acceptable, feasible, and appropriate despite the reticence of some to continue traditional practices such as smudging beyond the intervention time. The careful process of adapting the intervention to the Fort Peck Head Start teacher context was critical to the success of intervention fit for this population. Going forward the logistics of conducting the intervention at each of the Head Start program’s centers will need to be considered within the organizational climate to ensure a balance between organizational feasibility and acceptability and appropriateness for participants given the distinct culture of each Head Start center and dynamics between each center.

### Working with the community

Working within a CPBR framework was essential for the success of this intervention. We worked collaboratively with the Head Start program’s administration, the tribal council, and a tribal advisory board. Building a relationship between the research team and the community, participating in activities not directly related to our research such as teaching yoga and helping teachers become comfortable with online formats during the COVID-19 pandemic, helped to build trust and further develop comfort with an academic-community partnership.

The effectiveness of intervention delivery by a community-based interventionist contributes to the growing literature about the successful outcomes of delivering behavioral and psychological health support in remote areas where professional mental health services are few or underutilized [75, 76]. Using a paraprofessional embedded in the community enabled participants to receive culturally appropriate, competent psychological support that they otherwise would not have received. The facilitator also functioned as a cultural broker with knowledge of the community, and tribal and reservation history. The facilitator was able to respond to changing schedules and engage participants who lacked interest, time, or transportation in psychological care they otherwise would not have received. Evidence suggests that being from the community, being flexible and able to implement evidence-based interventions can contribute to individual and community level changes in health equity [75–77].

Utilizing mixed methods was beneficial to the study. If a quantitative analysis alone had been conducted, the positive effect of the intervention could have been missed. Qualitative data highlighted the practical significance of this intervention for participants and added depth and complexity to our quantitative findings. It also enabled corroboration of our data from different sources: observation, quantitative survey and qualitative focus groups. Our preliminary qualitative data revealed that teachers do not access existing psychological health support in their community. Observations of within session participation, the richness of the conversations, and the feedback post-intervention demonstrate that teachers benefited from time spent focused on their psychological health and well-being. Not every teacher embraced the importance of culture as a buffer from stress and support for well-being, but the safety of the forum (using a Native facilitator, conducting the sessions at each Head Start center, using a private room, not recording sessions as requested) and cultural sensitivity of the facilitator allowed all perspectives to be valued and heard. This supports community level group-based interventions as a successful method to provide culturally safe psychological care to underserved populations.

### Limitations

Results from this study must be considered within the context of its limitations. First our sample size was small (*n* = 18) limiting generalizability and power which might prevent finding meaningful associations between the intervention and a decrease in stress and improvement in well-being. However, we were working with a group of reservation based Native Americans who are part of a population often invisible in big data sets either because of low response rates to national surveys, by racial misclassification, or because they are subsumed into a broader category (e.g. “other”) which risks hiding specific disparities affecting that smaller population [78, 79]. By using mixed methods to capture the experiences of this relatively small population, we contribute important information that can be used to inform tribal, state, and federal planning as well as to inform the individuals and community itself of the need and right for culturally appropriate supportive psychological care. Furthermore, the use of mixed methods ensures participant experience and perspectives informed our quantitative results with the strengths of each approach compensating for their weaknesses [80].

Second there was no control group and therefore we cannot be sure that any changes – positive or negative – are due to the intervention itself. To test intervention effectiveness more rigorously, a true experimental design with randomization would be necessary. We did not randomize due to a small sample size and in a small community, both the reservation and Head Start, issues with cross over contamination and ethical issues of not including all who would like the support will have to be considered in the future.

By employing mixed methods our qualitative data allowed some explanation of the changes we found on the survey. Meeting with the teachers 3 months post intervention was an important time to present our findings and receive feedback on the overall study. These different time points, the use of observation, qualitative data and surveys help support the construct validity of this study.

All measures used on the survey were self-report which makes them susceptible to biases that may have undermined the validity of the study. Careful attention was paid to choosing reliable and valid measures that have been previously tested successfully or culturally adapted for Native American populations. Using mixed methods provided additional insight into the teachers’ experience that would not have been captured if the quantitative survey had been the only source of measurement.

Our intervention was 5 sessions planned for once a week over 5 weeks. However, because of extenuating circumstances including a major snowstorm that closed reservation program offices for a week and a tragic death in one of the research team’s families, it took 7 weeks to complete all sessions. This extended timeline may have moderated the interventions effect on outcome [81]. Flexibility on everyone’s part – including the Head Start administration, the intervention facilitator, and the participants who were all willing to adjust to these unexpected circumstances gives some confidence that the effect of these delays was minimal. As one participant noted, *“We are used to nothing going according to plan. It is a complicated life here on the rez.”* However, we cannot ignore that such breaks in intervention continuity could have influenced results. One consequence was that we had to combine focus groups at the completion of the study and observed that some teachers seeming more reluctant to speak. Ideally follow up individual interviews could have potentially overcome this barrier of speaking in the large group however lack of funding and time prevented this.

In terms of overall implementation success, (as opposed to participant/treatment effectiveness), we only measured AIM/IAM/FIM at the completion of the entire intervention rather than after each session. Had we measured AIM/IAM/FIM after each session, we would have gotten more specific information about each session rather than the overall intervention. However, observational data collected within sessions, the measurement of fidelity to intervention and noting dropout rates per session help support the positive results for implementation success. We hope that the data that we did collect will contribute to advancing the understanding of implementation processes and the assessment of implementation strategies particularly in low-resource communities and where cultural values and needs are a critical consideration.

Next steps for the program of research include designing a mixed methods pragmatic trial to determine the effectiveness of the intervention with Head Start teachers and potentially all Head Start staff across multiple reservations. This will enable the evaluation of treatment outcomes over time with the statistical power to detect the moderating effect of culture on stress. Therefore, we plan an active or wait-list control for the future effectiveness study.

## Conclusions

The results of this study indicate that a culturally informed intervention aimed at supporting the well-being of reservation-based Head Start teachers and buffering them from effects of stressors is feasible, acceptable, and appropriate with promising results. Using a culturally based intervention did promote discussions and awareness of the significance and importance of culture in the lives of participants. By delivering a culturally informed intervention, we highlighted the unique strengths and challenges facing reservation-based Native Americans. Given the complex traumas and disparities affecting Native Americans, it is critical that culture is an integral part of any intervention that is aimed at addressing the disparities they face.

Utilizing a group-based format facilitated by a paraprofessional embedded in the community provides a successful model for implementing psychological health care in a remote setting where access to specialized care is limited. Interventions that support the psychological health of Head Start teachers are few and this is one of the first to focus on Head Start teachers who serve Native American children and families.

Working collaboratively with the community to adapt and then implement the intervention has established a working partnership. Future directions include scaling the intervention into other reservation-based communities using an experimental design.

## Data Availability

The government-to-government relationship between the U.S. and federally recognized tribes acknowledges tribal sovereignty, tribal laws, and the tribes’ capacity to make decisions about research that is conducted within their jurisdictional boundaries. The Fort Peck tribal law that supports this research acknowledges the importance of community consent as well as individual consent and provided the particularities for data sharing as part of tribal research regulation. We have taken the necessary steps to ensure adherence to Fort Peck Tribal Law (Resolution #30-348-2020-03) and National Institutes of Health guidelines on sharing of data, in collaboration with our tribal partners, including seeking the appropriate tribal approvals to respect their tribal sovereignty and confidentiality. Requests for data from this study should be directed to Dr DH Wilson: Dwils103@jhu.edu.

## Abbreviations

ACE: Adverse Childhood Experiences
AIM: Acceptability of Intervention Measure
BCE: Benevolent Childhood Experiences
CD-RIS: Connor Davidsons Resilience Scale
CESD-10: Center for Epidemiological studies Depression Scale
CETA: Common Elements Treatment Approach
DSM-IV: Diagnostic and Statistical Manual of Mental Disorders
FIM: Feasibility of Intervention Measure
IAM: Intervention Appropriateness Measure
IHS: Indian Health Service
IRB: Institutional Review Board
OHS: Office of Head Start
RCT: Randomized Control Trial
SD: Standard Deviation
U.S.: United States
